# The Non-Immune RIP-k^b^ Mouse is a Useful Host for Islet Transplantation, as the Diabetes is Spontaneous, Mild and Predictable

**DOI:** 10.1080/15604280212530

**Published:** 2002

**Authors:** Robyn M. Sutherland, Joanne N. Mountford, Janette Allison, Leonard C. Harrison, Andrew M. Lew

**Affiliations:** 1 Walter and Eliza Hall Institute of Medical Research PO Royal Melbourne Hospital 3050 Australia; 2 The Department of Microbiology and Immunology University of Melbourne Parkville 3052 Australia

## Abstract

Chemically-induced diabetic mice and spontaneously diabetic NOD mice have been valuable as recipients for experimental islet transplantation. However, their maintenance often requires parenteral insulin. Diabetogenic chemicals can be cytotoxic to the host’s immune system and to other organs some of which are often used as the transplant site. Procurement of diabetic cohorts in the NOD mouse is problematic due to variability in the age of disease onset. We show that RIP-K^b^ mice, which spontaneously develop non-immune diabetes due to over-expression of the H-2K^b^ heavy chain in beta cells, offer many advantages as islet transplant recipients. Diabetes is predictable with a relatively narrow range of onset (4 wk) and blood glucose levels (23.0± 4.0 mmol/l for 39 males at 6 weeks of age). The diabetes is mild enough so that most diabetic mice can be maintained to 40 weeks of age without parenteral insulin. This consistency of diabetes avails that outcomes of intervention can be interpreted with confidence.


*Int. Jnl. Experimental Diab. Res.*, 3:37-45, 2002
© 2002 Taylor and Francis
1560-4284/02 $12.00 + .00

### The Non-Immune RIP-K$^b$ Mouse is a Useful Host for Islet Transplantation, as the Diabetes is Spontaneous, Mild and Predictable

ROBYN M. SUTHERLAND[a], JOANNE N. MOUNTFORD[a], JANETTE ALLISON[b], LEONARD C. HARRISON[a] AND ANDREW M. LEW[a]*

[a]Walter and Eliza Hall Institute of Medical Research, PO Royal Melbourne Hospital, Australia 3050

[b]The Department of Microbiology and Immunology, University of Melbourne, Parkville, Australia 3052

*Received: July 3, 2001; In final form:October 15, 2001*

**Chemically-induced diabetic mice and spontaneously diabetic NOD mice have been valuable as recipients for experimental islet transplantation. However, their maintenance often requires parenteral insulin. Diabetogenic chemicals can be cytotoxic to the host's immune system and to other organs some of which are often used as the transplant site. Procurement of diabetic cohorts in the NOD mouse is problematic due to variability in the age of disease onset. We show that RIP-K$^b$ mice, which spontaneously develop non-immune diabetes due to over-expression of the H-2K$^b$ heavy chain in beta cells, offer many advantages as islet transplant recipients. Diabetes is predictable with a relatively narrow range of onset (4 wk) and blood glucose levels (23.0$\pm$ 4.0 mmol/l for 39 males at 6 weeks of age). The diabetes is mild enough so that most diabetic mice can be maintained to 40 weeks of age without parenteral insulin. This consistency of diabetes avails that outcomes of intervention can be interpreted with confidence.**

*Keywords:* Islet, transplant, streptozotocin, alloxan, diabetes, NOD mouse

*Abbreviations:* RIP, rat insulin promoter

### INTRODUCTION

Mouse models of pancreatic islet transplantation often use recipients in which insulin-dependent diabetes has either been chemically induced with beta-cell cytotoxic agents, streptozotocin or alloxan, or allowed to develop

*Corresponding author: tel: (61 3) 9345 2555; FAX: (61 3) 9347 0852; e-mail: lew@wehi.edu.au

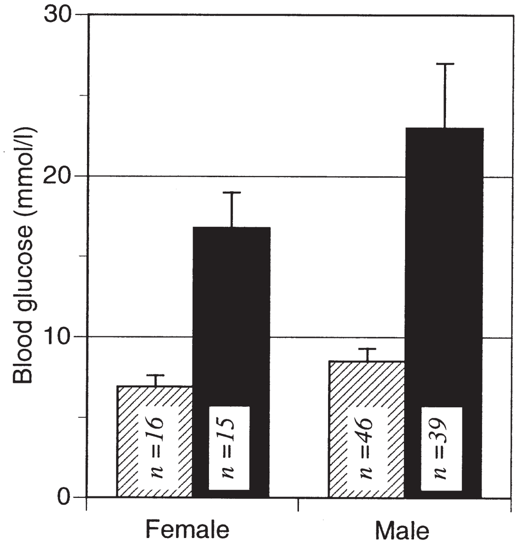

FIGURE 1
Blood glucose levels in female compared with male RIP-$K^b$ mice. The mean ± SD blood glucose level is shown for *n* non-diabetic (hatched bars, transgene negative) or diabetic (filled bars, transgene positive) female or male littermates at 6 weeks of age.

spontaneously as a result of autoimmune beta-cell destruction in the NOD mouse. These models are valuable, but have some disadvantages. Both chemical agents are hazardous, and streptozotocin is a carcinogen. The diabetogenic effect of these agents is dependent on multiple factors including mouse strain, sex, age and nutritional status eg. [1-8]. Their bioavailability can be variable due to their instability in solution [1, 9, 10]. As such, it can be difficult to control precisely the degree of induced beta-cell damage and consequent diabetes. In addition, the toxicity of streptozotocin and alloxan is not limited to the beta cell. Streptozotocin has direct effects on the liver [11], kidney [12] and immune system [13-16]. Likewise, alloxan is nephrotoxic [17] and possibly hepatotoxic [18]. These effects may compromise the interpretation of experimental outcomes.

Spontaneously diabetic NOD mice more closely resemble the clinical situation in humans and thus it could be argued are more appropriate recipients than chemically-induced diabetic mice. However, NOD mice are also not ideal. They present with diabetes over a wide time-span of months such that it is difficult to procure a large cohort of age-matched diabetic mice for transplant experiments. Once diabetic, they can only be maintained for 3-4 weeks before requiring insulin injections for survival. Finally, in studies where allo- or xeno-responses are being analyzed, it can be difficult to distinguish them from auto-immune responses to the graft.

A number of transgenic insulin-deficient diabetic mouse models have also been described (reviewed [19]), including several which are not immune-mediated [20-24]. However, none have been characterized with respect to their suitability as diabetic hosts for islet transplantation. One of these, the RIP-$K^b$ mouse, transgenically overexpresses the H-2$K^b$ class I heavy chain on the rat insulin promoter (RIP) in pancreatic beta-cells [20]. The mechanism of beta-cell damage by H-2$K^b$ has been proposed to result from the misfolding of the over-expressed heavy chain which somehow impairs the normal secretory pathways [25]. One RIP-$K^b$ lineage, designated 50-1, develops non-lethal diabetes, with mice surviving beyond 20 weeks of age. We postulated that these mice might obviate the need for iatrogenic intervention and develop diabetes with a reliable and predictable natural history. This should make them auspicious hosts for islet transplantation and interpretation of outcomes.

### MATERIALS AND METHODS

### *MICE*

CBA, B6.C-H2$^{bm1}$ (C57BL/6 with the bm1 mutation of H-2$K^b$) and C57BL/6-TgN(Rip-H2-$K^b$) (lineage 50-1,abbreviated RIP-$K^b$ [25])

mice were bred and maintained at the Walter and Eliza Hall Institute of Medical Research. Requests for RIP-$K^b$ mice should be addressed to Dr Andrew Lew at the Walter and Eliza Hall Institute. RIP-$K^b$ mice originated in CBA x B10.BR.F2 mice which were subsequently backcrossed to B6.C-$H2^{bm1}$ mice for 35 generations. Because we generally use CBA mice as pancreas graft recipients in our laboratory [26, 27], RIP-$K^b$ mice used in the current study were further backcrossed with CBA mice for at least 5 generations. All experimental procedures were approved by the Royal Melbourne Hospital Research Foundation Animal Ethics Committee.

### *Blood glucose measurements*

Non-fasting blood glucose was determined between 1-3 pm using Advantage blood glucose test strips (Roche, Castle Hill, Australia) and an Advantage Meter (Roche). A drop of blood was obtained via a glass capillary tube from the retro-orbital venous plexus of unanesthetised mice, or by tail bleed of anesthetised mice undergoing surgery. Mice with confirmed blood glucose measurements in excess of 11 mmol/l were classified as diabetic. Blood glucose levels are shown either for individual mice or as the mean ± SD for *n* mice.

### *Statistical analysis*

Differences between blood glucose levels or weights were analysed by two-tailed unpaired t tests using Prism v2.0a (GraphPad Software, Inc., San Diego, USA).

### *Pancreatic islet transplants*

Islet donors were 6-10 week old male and female CBA or B6.C-$H2^{bm1}$ mice. Pancreata were collagenase-digested, and islets were separated by density gradient centrifugation over Histopaque-1077 (Sigma, St Louis,USA) as described [28]. Islets were hand-picked and counted under an inverted microscope. Between 400-700 islets were injected, via a yellow micropipette tip, under the kidney capsule of 6-10 week old male RIP-$K^b$ recipients. Blood glucose levels were determined just prior to islet transplantation then monitored periodically as indicated. The graft plus kidney were recovered at the indicated times from mice which were either killed, or nephrectomized and allowed to recover before additional blood glucose determinations. The graft plus kidney were fixed in Bouin's solution before sectioning and staining with hematoxylin and eosin.

### RESULTS

### *Diabetes is more severe in male RIP-$K^b$ mice*

Diabetes incidence was compared in male and female mice (Figure 1). As expected, non-transgenic offspring do not develop diabetes. At six weeks of age diabetic male mice had a blood glucose of 23.0± 4.0 mmol/l compared to 16.8 ± 2.2 mmol/l in diabetic females ($p < 0.0001$). This rather tight standard deviation allows for reliable determination of any successful intervention. There was no overlap between diabetic and non-diabetic mice (Figure 2). Indeed, the difference in mean blood glucose between non-diabetic and diabetic mice at 6 weeks of age was 9.9 and 14.5 mmol/l for females and males respectively (Figure 1). This margin of difference should allow the use of mice of either sex as transplant recipients, although this margin is greater for male mice. Male recipients are also desirable in that donor pancreas of any sex can be transplanted without concern for H-Y antigen differences. This is particularly important for fetal tissue transplantation, as the sex may be unknown.

### *Diabetes in male RIP-$K^b$ mice develops early, is stable and does not require insulin injections*

A more comprehensive analysis of blood glu-

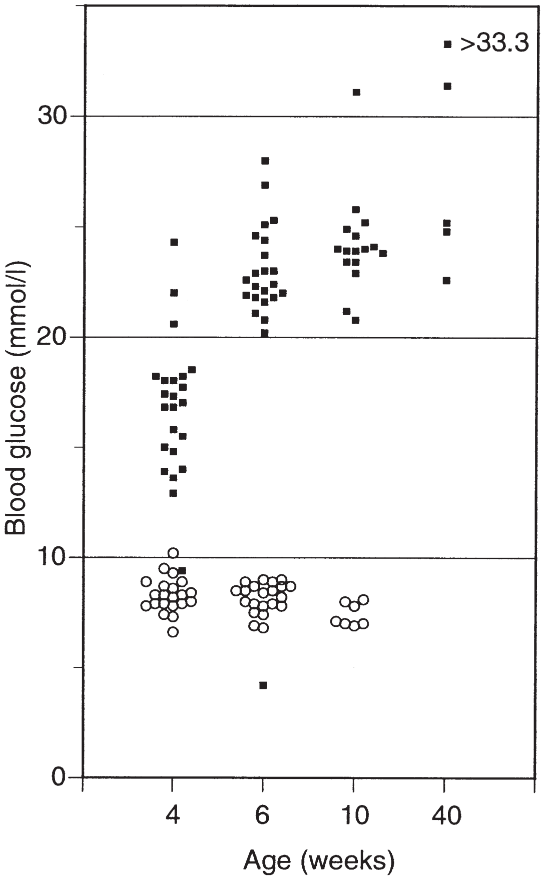

FIGURE 2
Blood glucose levels in male RIP-K$^b$ mice. Blood glucose levels are shown for individual diabetic (closed symbols, transgene positive ) and non-diabetic (open symbols, transgene negative) littermates. One mouse at 40 weeks of age had a blood glucose in excess of the 33.3 mmol/l limit of detection.

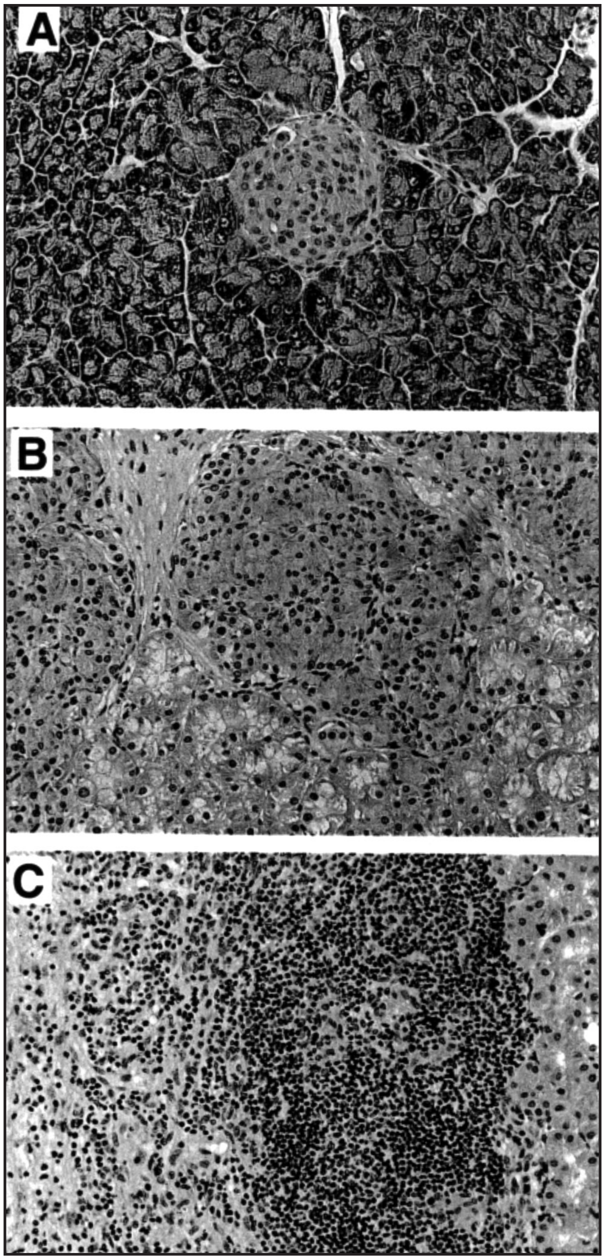

FIGURE 3
Hematoxylin and eosin stains (x200) of (A) the pancreas of a 13 week RIP-K$^b$ male, (B) a syngeneic CBA islet graft under the kidney capsule of a RIP-K$^b$ mouse, 98 days post-transplant, and (C) an allogeneic islet graft under the kidney capsule of a RIP-Kb mouse, 17 days post-transplant, showing islet destruction and residual mononuclear cell infiltrate.

cose levels at various ages was performed in a second cohort of male mice (Figure 2). Diabetes of moderate severity (16.8 $\pm$ 3.1 mmol/l) developed by 4 weeks of age in 96% of transgenic mice. The severity of diabetes increased by 6 weeks of age (22.2 $\pm$ 4.4 mmol/l) and blood glucose levels remained in excess of 20 mmol/l from 6 – 40 weeks. Five mice were followed out to 40 weeks of age. They continued to rear well, but were clearly smaller than non-diabetic littermates. One of these mice had a blood glucose above the limit of detection (33.3 mmol/l), was slightly hunched and had ruffled fur. All mice were killed at 40 weeks of age.

RIP-K$^b$ mice have delayed weight gain. The

TABLE 1 RIP-$K^b$ mice have delayed weight gain.

| Mouse age (weeks) | Diabetic | *n* | Blood glucose (mmol/l)* | Weight (g)* |
|---|---|---|---|---|
| 5 | - | 7 | 9.5 ± 1.3 | 18.7 ± 1.7 |
| | + | 7 | 19.8 ± 4.1 | 16.8 ± 1.3 |
| 15 | - | 5 | 7.8 ± 0.6 | 26.1 ± 2.6 |
| | + | 5 | 24.0 ± 3.7 | 23.0 ± 0.7 |
| 27 | - | 5 | 6.2 ± 0.5 | 29.1 ± 3.1 |
| | + | 5 | 22.5 ± 5.6 | 20.1 ± 1.7 |

*Mean ± SD for male mice development of diabetic compared to non-diabetic mice was assessed by weighing mice at 5, 15 and 27 weeks of age (Table 1). The weight of diabetic mice increased from 16.8 $\pm$ 1.3 g at 5 weeks to 23.0 $\pm$ 0.7 g at 15 weeks ($p$ < 0.0001), and at these times was only 10-12% less than non-diabetic littermates ($p$ < 0.05). Diabetic mice failed to gain weight between 15 and 27 weeks, and rather had some weight lost. The 27 week weight of 20.1 $\pm$ 1.7 g was 13% less than that of the same mice at 15 weeks (p<0.01), and 31% less than that of non-diabetic littermates (p<0.001). At 27 weeks, 4/5 diabetic mice appeared healthy; one was hunched, had ruffled fur and was killed.

### *RIP-$K^b$ MICE EXHIBIT NO EVIDENCE OF ISLET AUTOIMMUNITY BUT REJECT ALLOGENEIC PANCREATIC ISLET GRAFTS*

We next examined the suitability of RIP-$K^b$ mice for use as islet transplant recipients. The development of diabetes in RIP-$K^b$ mice is not immune-dependent: diabetes develops even if mice are neonatally-thymectomized [20] or are on an athymic nude mouse background [29]. In euthymic mice, diabetes develops in the absence of islet infiltration ([20], Figure 3A; a mouse

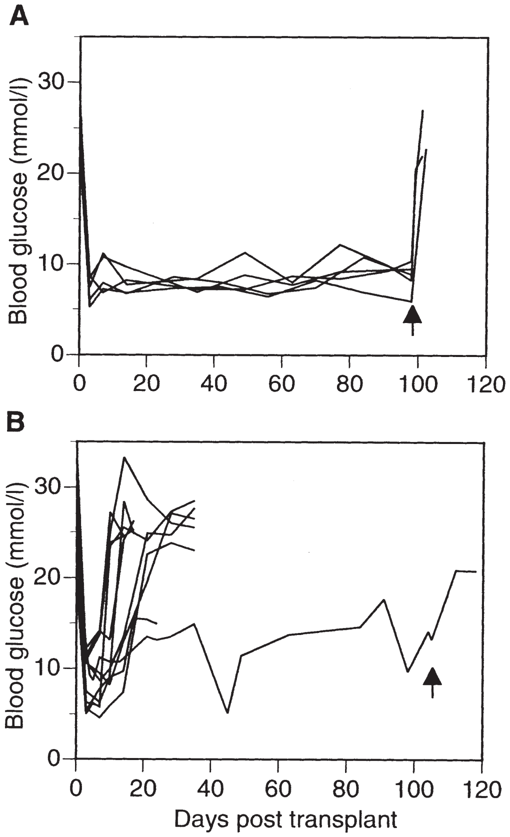

FIGURE 4
Blood glucose levels in RIP-$K^b$ recipients of (**A**) syngeneic CBA or (**B**) allogeneic B6.C-H2$^{bm1}$ islet grafts. Removal of the graft-bearing kidney by nephrectomy in 3/5 recipients of syngeneic and 1/11 recipients of allogeneic islet grafts is indicated by an arrow.

with blood glucose 25.3 mmol/l). The absence of an islet specific autoimmune response in the RIP-$K^b$ mice was further supported by the ability of syngeneic CBA islet transplants to reverse diabetes and maintain normoglycemia for the 98 day duration of the experiment (Figure 4A). Normoglycemia could be established by transplanting 400 islets. Transplants of fewer islets have not been attempted. Normoglycemia was reversed by nephrectomy of the graft-containing kidney (Figure 4A), indicating that the function of endogenous RIP-$K^b$ expressing islets remained impaired in the absence of hyperglycemic stress. The recovered syngeneic grafts contained numerous islets, but no immune infiltrate (Figure 3B).

Allogeneic islet transplants initially reversed diabetes in RIP-$K^b$ mice. In 9/11 mice the transplants were rapidly infiltrated by mononuclear cells and rejected (Figure 3C), such that blood glucose levels returned to pre-transplant levels by 21 days post-transplant (Figure 4B). In 2/11 mice, the response was less vigorous. These mice had numerous intact islets surrounded by peri-islet mononuclear infiltrates when grafts were recovered 24 and 104 days post-transplant. Mild diabetes (around 14 mmol/l) at the time of graft recovery was suggestive of impaired islet function (Figure 4B). Graft recovery at 104 days post-transplant by nephrectomy resulted, as for syngeneic grafts, in a return of the blood glucose to the pre-transplant level (20.1 mmol/l).

### DISCUSSION

RIP-$K^b$ mice develop a spontaneous, non-immune diabetes as a consequence of overexpression of the H-2$K^b$ heavy chain under the control of RIP [20]. It has been proposed that the H-2$K^b$ heavy chain is misfolded in the absence of $\beta_2$M light chain, and accumulates in insulin secretory granules thereby disrupting the processing and secretion of insulin [25]. Indeed, $\beta_2$M expressed under the control of RIP can be detected in the insulin secretory granules, and its coexpression with the H-2$K^b$ heavy chain reduces the severity of RIP-$K^b$ diabetes [25]. Transgenic expression of various other molecules including class II MHC molecules [21, 22], calmodulin [24], and H-ras [23], under the control of the insulin promoter can also result in non-immune mediated diabetes. However, it remains moot whether any of these mice will be suitable as diabetic hosts for islet transplantation. Some are clearly not. For example, the RIP-ras mice develop diabetes late (5 months), become severely diabetic and die within a few weeks [23]. The RIP-calmodulin mice develop diabetes within hours of birth and at least some lines suffer from fertility problems [24]. The current study has shown that the RIP-$K^b$ class I heavy chain transgenic mouse has several characteristics that are desirable in recipients of allo- and xeno-geneic islet transplants. Diabetes develops early and in a predictable and uniform fashion such that by 6 weeks of age male mice routinely have a blood glucose level of between 20-28 mmol/l. Consequently, groups of mice matched for age, sex and degree of diabetes can be acquired with ease. In practical terms, mice can be screened at 4 weeks of age: there are no false positives, while false negatives (due to incomplete expression of the diabetic phenotype at this early time-point) are rare and unimportant as they are discarded. By contrast, diabetes onset in NOD mice is relatively late and in our colony varies from 14 - 28 weeks of age, which does not facilitate acquisition of diabetic cohorts. The production of diabetic groups can also be problematic with streptozotocin or alloxan, because even when mouse strain, age and sex are matched, the diabetogenic effect is variable. This can be addressed in part by determining the appropriate dose for a given mouse strain and weighing individual mice prior to injection.

However, other factors such as drug instability are not so easily controlled. Both streptozotocin and alloxan have short half-lives in aqueous solution and plasma (reviewed [1]). The effective dose of streptozotocin is dependent on the ratio of alpha and beta anomers [9], which varies between manufacturers, batches and time in solution [10, 30]. Clearly, diabetes induced by streptozotocin or alloxan entails more variables, is less predictable and is more labour intensive than in the RIP-$K^b$ model. Streptozotocin and alloxan are also toxic and, in the case of streptozotocin, carcinogenic, necessitating appropriate safety measures to protect researchers and animal technicians.

The general toxicity of diabetogenic drugs may influence graft survival and confound interpretation of results. Islets are usually grafted under the kidney capsule of mice, and less often into the liver, spleen or testes. Notably, the liver is a clinically relevant graft site in humans [31]. Streptozotocin can alter liver morphology and impairs liver function [11]. Nephrotoxic effects have also been described [12]. Alloxan causes atrophy of kidney tubules and interstitial nephritis [17], and is a potential liver toxin [18]. While it is unknown how such effects impact on graft outcome, an advantage of RIP-$K^b$ mice is that pathologies at the graft site are limited to the secondary effects of diabetes and the graft procedure itself.

Streptozotocin is also directly toxic to thymocytes and bone marrow cells [13-15], and mutagenic for splenocytes due to its capacity to induce DNA strand breaks [16]. The functional consequences of streptozotocin treatment include suppression of cell-mediated immune responses independent of hyperglycemia [13, 14, 16]. Thus, with streptozotocin it may be difficult to attribute outcomes to an experimental immunosuppressive regimen. Similarly, the abnormal immune system of NOD mice may influence the nature of graft rejection. Preactivated autoimmune effectors present in diabetic NOD mice may dominate the emerging allo- or xeno-response and thus confound analysis. No such immune perturbations occur in the RIP-$K^b$ mouse. Delayed rejection of allogeneic B6.C-$H2^{bm1}$ islets occurred in 2/11 RIP-$K^b$ mice, but was not considered indicative of an immunocompromised state. In these two cases the host RIP-$K^b$ mice had been backcrossed to CBA mice for 5 - 6 generations, and we consider it likely that they retained some parental B6.C-$H2^{bm1}$ histocompatibility genes. More recent B6.C-$H2^{bm1}$ transplants into RIP-$K^b$ mice, backcrossed for 9 - 10 generations, have been uniformly rejected by day 21 post transplant.

Spontaneous reversal of diabetes due to beta-cell neoplasia or neogenesis can occur, when using streptozotocin or alloxan induced diabetic hosts [3, 32-35]. Thus, it can be difficult to determine whether reversal of diabetes is due to this or to the grafting procedure. Hence, it is desirable to confirm graft function at the conclusion of an experiment, for instance by nephrectomy of a graft bearing kidney and reversion to hyperglycemia. Such confirmation is not feasible when grafting into sites such as the liver since the graft cannot be removed without the death of the host. Our experience in RIP-$K^b$ mice shows that diabetes is stable with increasing blood glucose with age and the loss of host beta-cell function is not reversible even when hyperglycemic stress is relieved by syngeneic islet grafts. Logically, even if neogenesis of host beta cells does occur, the function of the neo-beta cells should be impaired by expression of the RIP-$K^b$ transgene. Furthermore, the RIP-$K^b$ mouse has no predilection to beta-cell neoplasia unlike models using mutagenic reagents such as streptozotocin. Thus, we propose that reversal of hyperglycemia in RIP-$K^b$ mice can be more reliably attributed to the graft than in alloxan or streptozotocin treated diabetic mice.

The long-term survival of diabetic mice without the need for insulin therapy is a distinct advantage when grafting stem cell or fetal tissue grafts that do not result in immediate reversal of diabetes. NOD mice require insulin treatment within 3-4 weeks of onset of diabetes in order to survive, while chemically-induced diabetic mice may require immediate insulin treatment. All RIP-$K^b$ mice can reliably be maintained without parenteral insulin for >20 weeks after the onset of diabetes; the vast majority even longer. Despite blood glucose levels in excess of 20 mmol/l from 6 weeks of age, they generally remain healthy although they do not grow as rapidly as wild type mice. By 27 weeks the occasional mouse shows signs of discomfort (hunching, ruffling of fur, reduced activity) and must be killed, but it is possible to maintain most mice out to 40 weeks of age. If mice are transplanted at 6 weeks of age, this provides a period of up to 34 weeks during which grafts can mature *in vivo*. If glucose toxicity for grafted tissue is a consideration, the stable hyperglycemia in the RIP-$K^b$ mouse, should be more readily normalized than hyperglycemia in NOD or chemically-induced diabetic mice. Furthermore, the stability of RIP-$K^b$ diabetes means that in the absence of parenteral insulin, even partial reductions in blood glucose, eg. from >20 to about 14 mmol/l in the case of allografts with peri-islet infiltration in the current study, can be interpreted with confidence as evidence of graft function.

It is clear that as pancreatic islet transplant recipients RIP-$K^b$ mice offer advantages with respect to ease of use, stability of diabetes, lack of autoimmune effects, and lack of iatrogenic effects (from injecting cytotoxic agents, or insulin). The introduction of the RIP-$K^b$ model is particularly timely in view of current intense interest in the transplant of stem cells and fetal pig tissue, and transplants into the liver. It will be of additional interest to determine if RIP-$K^b$ mice are a useful model for studying chronic hyperglycemia and potential diabetic complications.

*ACKNOWLEDGEMENTS*

This work was supported by a Program grant from the Juvenile Diabetes Foundation International- National Institutes of Health, and by grants from Diabetes Australia and the National Health and Medical Research Council of Australia.

We are grateful to Michelle Latimer for expert technical assistance with mice, and Prof. Tom Mandel for critical reading of the manuscript.

### REFERENCES

[1] Rerup, C.C. (1970) Drugs producing diabetes through damage of the insulin secreting cells, *Pharmacol Rev*, **22**, 485-518.

[2] Rossini, A.A., Appel, M.C., Williams, R.M. and Like, A.A. (1977) Genetic influence of the streptozotocin-induced insulitis and hyperglycemia, *Diabetes*, **26**, 916-920.

[3] Cohn, J.A. and Cerami, A. (1979) The influence of genetic background on the susceptibility of mice to diabetes induced by alloxan and on recovery from alloxan diabetes, *Diabetologia*, **17**, 187-191.

[4] Maclaren, N.K., Neufeld, M., McLaughlin, J.V. and Taylor, G. (1980) Androgen sensitization of streptozotocin-induced diabetes in mice, *Diabetes*, **29**, 710-716.

[5] Riley, W.J., McConnell, T.J., Maclaren, N.K., McLaughlin, J.V. and Taylor, G. (1981) The diabetogenic effects of streptozotocin in mice are prolonged and inversely related to age, *Diabetes*, **30**, 718-723.

[6] Wolf, J., Lilly, F. and Shin, S.I. (1984) The influence of genetic background on the susceptibility of inbred mice to streptozotocin-induced diabetes, *Diabetes*, **33**, 567-571.

[7] Eizirik, D.L. and Migliorini, R.H. (1984) Reduced diabetogenic effect of streptozotocin in rats previously adapted to a high-protein, carbohydrate-free diet, *Diabetes*, **33**, 383-388.

[8] Wright, J.R., Jr. and Lacy, P.E. (1988) Synergistic effects of adjuvants, endotoxin, and fasting on induction of diabetes with multiple low doses of streptozocin in rats, *Diabetes*, **37**, 112-118.

[9] Rossini, A.A., Like, A.A., Dulin, W.E. and Cahill, G.F., Jr. (1977) Pancreatic beta cell toxicity by streptozotocin anomers, *Diabetes*, **26**, 1120-1124.

[10] Oles, P.J. (1978) High-pressure liquid chromatographic separation and determination of anomeric forms of streptozocin in a powder formulation, *J Pharm Sci*, **67**, 1300-1302.

[11] Carnovale, C.E. and Rodriguez Garay, E.A. (1984) Reversible impairment of hepatobiliary function induced by streptozotocin in the rat, *Experientia*, **40**, 248-250.

[12] Levine, B.S., Henry, M.C., Port, C.D. and Rosen, E. (1980) Toxicologic evaluation of streptozotocin (NSC 85998) in mice, dogs and monkeys, *Drug Chem Toxicol,* **3,** 201-212.

[13] Nichols, W.K., Spellman, J.B., Vann, L.L. and Daynes, R.A. (1979) Immune responses of diabetic animals. Direct immunosuppressant effects of streptozotocin in mice, *Diabetologia,* **16,** 51-57.

[14] Nichols, W.K., Vann, L.L. and Spellman, J.B. (1981) Streptozotocin effects on T lymphocytes and bone marrow cells, *Clin Exp Immunol,* **46,** 627-632.

[15] Wellhausen, S.R. (1986) Definition of streptozocin toxicity for primary lymphoidal tissues, *Diabetes,* **35,** 1404-1411.

[16] Gaulton, G.N., Schwartz, J.L. and Eardley, D.D. (1985) Assessment of the diabetogenic drugs alloxan and streptozotocin as models for the study of immune defects in diabetic mice, *Diabetologia,* **28,** 769-775.

[17] Evan, A.P., Mong, S.A., Connors, B.A., Aronoff, G.R. and Luft, F.C. (1984) The effect of alloxan, and alloxan-induced diabetes on the kidney, *Anat Rec,* **208,** 33-47.

[18] Harman, A.W. and Fischer, L.J. (1982) Alloxan toxicity in isolated rat hepatocytes and protection by sugars, *Biochem Pharmacol,* **31,** 3731-3736.

[19] Harrison, L.C., Campbell, I.L., Allison, J. and Miller, J.F. (1989) MHC molecules and beta-cell destruction. Immune and nonimmune mechanisms, *Diabetes*, **38,** 815-818.

[20] Allison, J., Campbell, I.L., Morahan, G., Mandel, T.E., Harrison, L.C. and Miller, J.F. (1988) Diabetes in transgenic mice resulting from over-expression of class I histocompatibility molecules in pancreatic beta cells, *Nature*, **333,** 529-533.

[21] Sarvetnick, N., Liggitt, D., Pitts, S.L., Hansen, S.E. and Stewart, T.A. (1988) Insulin-dependent diabetes mellitus induced in transgenic mice by ectopic expression of class II MHC and interferon-gamma, *Cell*, **52,** 773-782.

[22] Lo, D., Burkly, L.C., Widera, G., Cowing, C., Flavell, R.A., Palmiter, R.D. and Brinster, R.L. (1988) Diabetes and tolerance in transgenic mice expressing class II MHC molecules in pancreatic beta cells, *Cell,* **53,** 159-168.

[23] Efrat, S., Fleischer, N. and Hanahan, D. (1990) Diabetes induced in male transgenic mice by expression of human H-ras oncoprotein in pancreatic beta cells, *Mol Cell Biol,* **10,** 1779-1783.

[24] Epstein, P.N., Overbeek, P.A. and Means, A.R. (1989) Calmodulin-induced early-onset diabetes in transgenic mice, *Cell,* **58,** 1067-1073.

[25] Allison, J., Malcolm, L., Culvenor, J., Bartholomeusz, R.K., Holmberg, K. and Miller, J.F. (1991) Overexpression of beta 2-microglobulin in transgenic mouse islet beta cells results in defective insulin secretion, *Proc Natl Acad Sci USA,* **88,** 2070-2074.

[26] Zhan, Y., Martin, R.M., Sutherland, R.M., Brady, J.L. and Lew, A.M. (2000) Local production of anti-CD4 antibody by transgenic allogeneic grafts affords partial protection, *Transplantation*, **70,** 947-954.

[27] Sutherland, R.M., Brady, J.L., Georgiou, H.M., Thomas, H.E. and Lew, A.M. (2000) Protective effect of CTLA4Ig secreted by transgenic fetal pancreas allografts, *Transplantation,* **69,** 1806-1812.

[28] Liu, M. and Shapiro, M.E. (1995) A new method for isolation of murine islets with markedly improved yields, *Transplant Proc,* **27,** 3208-3210.

[29] Mandel, T.E., Allison, J., Campbell, I.L., Koulmanda, M., Malcolm, L., Cutri, A. and Miller, J.F. (1991) Inherent beta-cell dysfunction induced by transgenic expression of allogeneic major histocompatibility complex class I antigen in islet cells, *Autoimmunity*, **9,** 47-53.

[30] Povoski, S.P., McCullough, P.J., Zhou, W. and Bell, R.H., Jr. (1993) Induction of diabetes mellitus in Syrian golden hamsters using stored equilibrium solutions of streptozotocin, *Lab Anim Sci,* **43,** 310-314.

[31] Shapiro, A.M., Lakey, J.R., Ryan, E.A., Korbutt, G.S., Toth, E., Warnock, G.L., Kneteman, N.M. and Rajotte, R.V. (2000) Islet transplantation in seven patients with type 1 diabetes mellitus using a glucocorticoid-free immunosuppressive regimen, *New Engl J Med,* **343,** 230-238.

[32] Kazumi, T., Yoshino, G., Fujii, S. and Baba, S. (1978) Tumorigenic action of streptozotocin on the pancreas and kidney in male Wistar rats, *Cancer Res,* **38,** 2144-2147.

[33] Hartmann, K., Besch, W. and Zuhlke, H. (1989) Spontaneous recovery of streptozotocin diabetes in mice, *Exp Clin Endocrinol,* **93,** 225-230.

[34] Fernandes, A., King, L.C., Guz, Y., Stein, R., Wright, C.V. and Teitelman, G. (1997) Differentiation of new insulin-producing cells is induced by injury in adult pancreatic islets, *Endocrinology,* **138,** 1750-1762.

[35] Su, E.N., Alder, V.A., Yu, D.Y., Yu, P.K., Cringle, S.J. and Yogesan, K. (2000) Continued progression of retinopathy despite spontaneous recovery to normoglycemia in a long-term study of streptozotocin-induced diabetes in rats, *Graefes Arch Clin Exp Ophthalmol,* **238,** 163-173.

